# Adaptation of Fracture Mechanics Methods for Quality Assessment of Tungsten Carbide Cutting Inserts

**DOI:** 10.3390/ma14133441

**Published:** 2021-06-22

**Authors:** Sylwester Samborski, Jerzy Józwik, Jakub Skoczylas, Mariusz Kłonica

**Affiliations:** Faculty of Mechanical Engineering, Lublin University of Technology, Nadbystrzycka 36, 20-618 Lublin, Poland; j.jozwik@pollub.pl (J.J.); j.skoczylas@pollub.pl (J.S.); m.klonica@pollub.pl (M.K.)

**Keywords:** tungsten carbide, fracture toughness, Charpy impact test, three-point bending test, fractography

## Abstract

Tungsten carbide (WC) is well known as one of the hardest materials widely used in machining, cutting and drilling, especially for cutting tools production. Knowing fracture toughness grants the opportunity to prevent catastrophic wear of a tool. Moreover, fracture toughness of WC-based materials may vary because of different material compositions, as well as a different way of production. Hence, each material should be treated individually. In this paper, SM25T (HW) tungsten carbide (HW—uncoated grade, TNMR 401060 SM25T, manufactured by Baildonit company, Katowice, Poland) was taken into consideration. Sintered carbides—designated as S—are designed to be applied for machining steel, cast steel and malleable cast iron. Fracture mechanics methods were adapted to make a quality assessment of WC cutting inserts. Both quasi-statical three-point bending tests, as well as Charpy dynamic impact tests, were performed to calculate static and dynamic fracture toughness (*K*_IC_ and *K*_ID_, respectively). In addition, a special emphasis was placed on the microscopic analysis of fracture surfaces after impact tests to discuss material irregularities, such as porosity, cracks and so-called “river patterns”. There is a lack of scientific works in this field of study. However, cutting engineers are interested in obtaining the experimental results of that kind. Although there are a few standardized methods that may be used to determine fracture toughness of hard metals, none of them is expected to be the most reliable. Moreover, there is a lack of scientific works in the field of determining static and dynamic fracture toughness of WC by the presented method. The proposed examination solution can be then successfully used to calculate toughness properties of WC-based materials, as the results obtained seem to be with a good agreement with other works.

## 1. Introduction

Wear of cutting edges occurs due to the exploitation process of cutting tools and leads to loss of its machining properties. Durability of cutting edges affects the costs of production because of additional expenditures such as a cost of worn tools and their regeneration, as well as the costs of technological machine downtime caused by necessity of tool replacement, etc. Besides, tool life of cutting edges (cutting plates) is strongly influenced by the following: machined material, cutting edge material, cutting parameters and a type of cutting oil. There are some materials, such as titanium alloys and Inconel alloys (e.g., 625 or 718 type), the case of which the machining is connected with extremally fast tool wear. Generally, wear of cutting edge has a negative impact on machining. If the cutting path rises due to the wearing of cutting edges, then the cutting forces and their amplitudes also rise. Nowadays, so-called integral components have been produced, especially in the space and aerospace industries. They are designed to be a good substitution of sets consisting of several dozen or several hundred parts. Integral components have complex construction and production, requiring removing several dozen percentage points of semi-finished product (often over 50% of semi-finished product). Damage of cutting tools (cutting plate) during the machining of integral components leads to their failure, especially in the case of thin-walled parts applied in the space and aerospace industries. Therefore, from the technological and economical point of view it is important to define and better understand the materials used for producing of cutting tools (cutting plates), especially if there are dynamic changes occurring while machining.

Tungsten (W) is one of the most attractive materials [1,2,3,4]. However, tungsten is known as a material which properties are strongly influenced by its fabrication techniques [5] and anisotropy of its microstructure [6], as well as a grain size of the structure surface [7]. Nogami et al. proved that Charpy impact properties of W depend on the above-mentioned material attributes [5]. Furthermore, Kuczmaszewski et al. examined the impact of carbide grain size in end mills on a tool life of cutting edge during milling of Ti6A14V titanium alloy parts. They proved that ultra-fine grain milling cutters can resist chipping better than coarse grain milling cutters. Moreover, in the case of ultra-fine milling cutters roughness parameters of machined surfaces were at a lower level than in the case of coarse-grain milling cutters [8].

Tungsten carbide (WC) is an inorganic compound that consists of both tungsten (W) and carbon (C). WC is one of the hardest metals, harder and more durable and resistant than standalone tungsten. WC is applied in various fields of manufacturing, e.g., machining, metal cutting and drilling in different branches of engineering, including aerospace and automotive [9,10]. It has very high hardness as well as wear toughness, even at high temperature such as 700–1000 °C. Other important WC features are as follows: very good hardness, high electrical conductivity, good thermal properties and high melting point [10]. Furthermore, materials described as “cemented carbide” are often based on applying WC as a component. This type of materials refers to composites produced using the powder metallurgy process by cementing the carbide grains into a matrix [9]. It is expected that fracture toughness of cemented WC would be higher than of pure WC, which is a ceramic that is characterized as the one with a brittle nature regardless of its high hardness [11]. Parihar et al. analyzed the static fracture toughness (*K*_IC_) of cobalt-bonded WC and showed that its values were equal to approx. 7–13 MPa·m^1/2^ and were strongly influenced by sintering parameters [12]. Static fracture toughness of similar materials tested by Li [11] was in a range of approx. 5.5–7.5 MPa·m^1/2^ and it was proved that the values were affected by sintering duration. However, static fracture toughness of ferrous cemented WC was experimentally defined to be lower than 4.5 MPa·m^1/2^ [13]. Moreover, Wang et al. showed that static fracture toughness of WC-CoCr coatings was equal to approx. 4.97–7.12 MPa·m^1/2^ and depended on different WC grain characteristics [14]. Lamberson compared the values of static and dynamic fracture toughness (*K*_ID_) for cemented WC carbides. Static fracture toughness was equal to approximately 8–13 MPa·m^1/2^ while the dynamic reached higher average values at a level of approx. 16–21 Mpa·m^1/2^, depending on grade size and specimen type [15]. Based on [16,17] fracture toughness of WC generally increases with increasing grain size, as well as with increasing Co content. Because there is no specific standardized method designated to determine fracture toughness of hard metals various methods are used that might cause differences in fracture toughness values, depending on the selected method.

Most of the machining tools produced of carbides is made of recycled carbides. In recent years, the need for recycling is growing because of decreasing number of raw material resources. In the case of tungsten, its deposits are evaluated at 7 million tons, which is expected to be enough for 100 years of exploitation. Carbides are recycled mainly from worn plates and drills which are sold by former users. It is worth to notice that production of tools based on using recycling carbide need 70% less energy than in the case of using extracted resources. Moreover, the amount of carbon dioxide emitted can be limited at 40%.

In the literature several papers regarding WC-based materials fracture toughness can be found but many of them are based on computing static fracture toughness by using methods different than the ones which are the point of this study. A few standardized methods have been used so far to determine fracture toughness of WC-based materials, such as using Vickers indenter [11,12,13,14], three-point bending test [18], four-point bending test [19], double cantilever beam test [19], double torsion test [20], including pre-crack employment [17,21]. However, none of the above mentioned methods is expected to be the most reliable. In addition, there is a lack of publications regarding static and dynamic fracture toughness calculations based on mechanical strength tests, i.e., three-point bending test and Charpy impact test, conducted with accordance to the methods and formulae further described in Section 2. However, the presented method is widely used in the case of other materials, such as ceramics and epoxies, so the main purpose of the study was to check if this technique can be also successfully applied for testing fracture toughness of hard metals. Moreover, there are many papers regarding cemented WC in various configurations whereas there is limited number of studies regarding specific WC types, such as SM25T which was taken into consideration in the paper. Nowadays, there is a growing demand on providing this kind of results, because defining a phenomena that can occur during material fracture as well as calculating static and dynamic fracture toughness can give an opportunity to improve carbide attributes by modifying its composition and letting it define critical machining parameters to prevent catastrophic wear of a cutting edge. It is important especially in the case of machining parts with discontinuous geometric structure (parts with notch, interior cracks, grooves, keyways, splines, pores and empty spaces) and heterogenous interior structure (irregularities with high hardness, locally tempered parts, hybrid-material parts, welded or riveted parts). All above mentioned parts can cause dynamic-impact effect on tool life. Hence, obtaining static and dynamic fracture toughness for the WC specimens will give the opportunity to compare the results with other tool materials as well as enrich the scientific knowledge in the field of material behavior and its catastrophic failure toughness. Recently, WCs are subjected to cutting processes [22,23,24,25]. Thus, defining fracture toughness parameters allow for optimizing the force and torque values while fixing and optimal points of support for the machined WC. This could be also helpful for selection of proper tools and technological parameters of machining. Experimental methods and equipment used in the paper to determine fracture toughness values have been already successfully applied in the case of other materials [26,27,28].

## 2. Experimental Procedure

SM25T tungsten carbide was used during the examination. According to the information given by its manufacturer (Baildonit company, Katowice, Poland) sintered carbides designated as S—are designed to be applied for machining steel, cast steel and malleable cast iron. 25 corresponds to the range of application groups according to ISO [29]. Chemical composition of SM25T is as follows: 69.5% of WC, 21% of total content of TiC, TaC, NbC, 9.5% of Co. Average grain size is equal to 1–2 µm, density 12.6 g/cm^3^, transverse rapture strength 2000 MPa, hardness 1550 HV. This kind of WC is widely applied for production of cutting tools, e.g., coated and uncoated cutting plates with multiple cutting edges (HW—uncoated grade, TNMR 401060 SM25T, manufactured by Baildonit company, Katowice, Poland). SM25T (HW) is a type of carbide used for chipping machining of steels, cast steels and stainless steels in demanding environmental conditions. According to catalogue information this material is well known as the one with high dynamic fracture toughness. Thus, it can be successfully applied in the case of large section cutting layers at high and moderate cutting speed. One of the typical applications of SM25T (HW) is machining of wheel sets. Furthermore, tools made of this carbide can be used for machining the following materials: steels in P15–P40 range: structural carbon steel for general purposes, low alloy steel (annealed or tempered steel), high speed steel (annealed), cast steel (no alloy or low alloy) and stainless steels in M25–M35 range: ferrite-martensite steels, precipitation hardened steels, austenite steels. Hardness of a material which can be machined by using this kind of WC should be over 135 HB and up to 250 HB.

**Figure 1:**
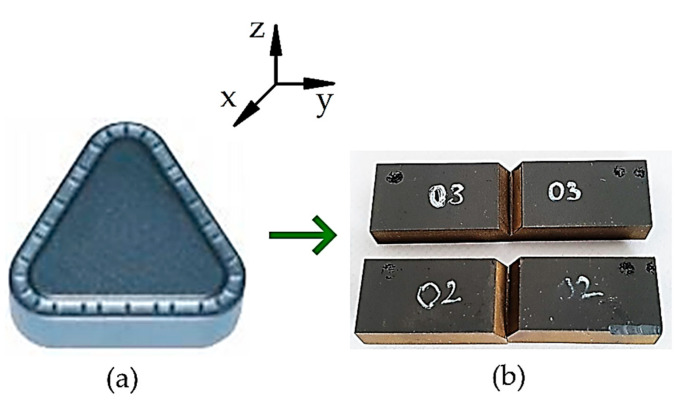
Specimen preparation: (**a**) Cutting plate made of SM25T (TNMR 401060 SM25T) tungsten carbide, produced by Baildonit company; (**b**) real view of specimens used for testing.

Specimens were prepared by erosive cutting using SL600Q cutting machine (Sodick Co., Ltd., Fukui, Japan). The example of cutting plate made of SM25T (TNMR 401060 SM25T) tungsten carbide used for specimen preparation and real view of two selected specimens used for testing are presented in Figure 1a,b, respectively. The limited number of samples is caused mainly by the high material cost and the technical difficulties while cutting. Moreover, the purpose of the study was to recognize the possibility for applying the specific procedures described in [30] to solve the following problems of WC plates: wear, toughness, quality check etc.

**Figure 2:**
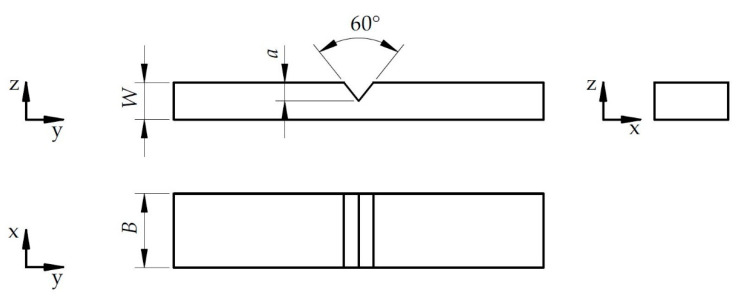
Geometrical parameters of WC specimen, where: *B*—thickness, *W*—width, *a*—notch length.

Geometrical parameters of WC specimen used for testing is presented in Figure 2. Sets of axes indicated in Figure 1 and Figure 2 show the direction towards which the specimens were cut. Each sample had thickness *B* = 10 mm, width *W* = 5 mm and notch cut with the length *a* = 2.5 mm. It should be noticed that the samples were prepared without pre-cracking which can influence on the stress concentration at the beginning of crack propagation, as well as on the measurements.

Two types of strength tests, static and dynamic, were conducted on tungsten carbide (WC) specimens: a three-point bending test as well as an instrumented impact test. Four specimens were prepared, two for each test type.

In the study, the mechanical behavior of tungsten carbide under static and dynamic loading was examined. The following formulae were used to calculate values of static fracture toughness (*K*_IC_) and dynamic fracture toughness (*K*_ID_) [30,31,32,33,34]:

(1) KIC=Ps MAXSsBW32ζ(aW) [MPam]

(2) KID=Pd MAXSdBW32ζ(aW) [MPam]

(3) ζ(aW)=1.5(aW)12{1.99−aW(1−aW)[2.15−3.93aW+2.7(aW)2]}(1+2aW)(1−aW)32

where *P*_s MAX_ is the mean value of maximal static force, *P*_d MAX_ is the mean value of maximal dynamic force read from its time-course; *S*_s_ and *S*_d_ is a span of supporting rollers in a three-point bending machine and a span of Charpy pendulum buttresses, respectively; *B* and *W* are sample’s dimensions; *ξ*(*a/W*) is the function of notch dimensions and *a/W* is a normalized depth of the notch. Using the same formulae to calculate both static and dynamic fracture toughness required that the dynamic loading criterion was fulfilled as the necessary condition [30,32,33]. Hence, the criterion was fulfilled during the impact tests because the dynamic forces oscillations faded in time.

The tests took place at ambient conditions. Dynamic tests were performed using the instrumented Charpy pendulum. The machine was fabricated by KB Prueftechnik GmbH (Hochdorf–Assenheim, Germany) and was equipped with a 7.5 J tup which had a fall angle and a maximum velocity equal to 157.32° and 3.815 m/s, respectively. Force, time, energy as well as displacement were acquired by A/D PC card NuDAQ PCI-9812 (AdLink Technology Inc., Taiwan, China). The time-course of the impact force was registered with a frequency of 1 MHz by a sensor just when the tup met the sample surface. The span was set to 40 mm.

The Autograph AGS-X 5 kN universal testing machine manufactured by Shimadzu Corporation (Kyoto, Japan) was used to conduct the three-point bending tests. The specimens were loaded quasi-statically with loading crosshead’s speed set to 1 mm/min. The three-point bending fixture used in the experiment was the MTS bending fixture (model no. 642.01A) with the bottom rolls’ diameter equal 5 mm; the loading roll had a diameter of 10 mm. This fixture follows all the necessary standards and enables both three- and four-point bending with adjustable span. The span of the supporting rollers was equal to 25 mm. Following parameters were stored on PC’s hard drive: force, time and displacement.

The surface structure and its parameters were measured using Alicona Infine Focus, optical 3D measurement system, which is based on the technology of focus variation and let to conduct advanced measurements of geometry, profile as well as surface topography. The system has a high vertical resolution (up to 10 nm, depends on the lens) which helps to measure cutting tools wear and control parts produced in different branches of industry such as automotive, aerospace and medicine.

## 3. Results and Discussion

**Figure 3:**
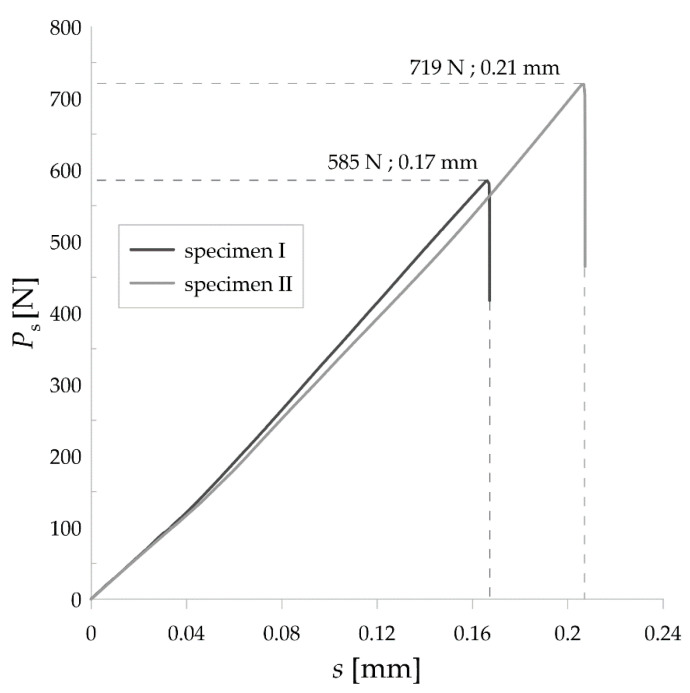
Static force versus displacement for the two tested SM25T specimens.

**Figure 4:**
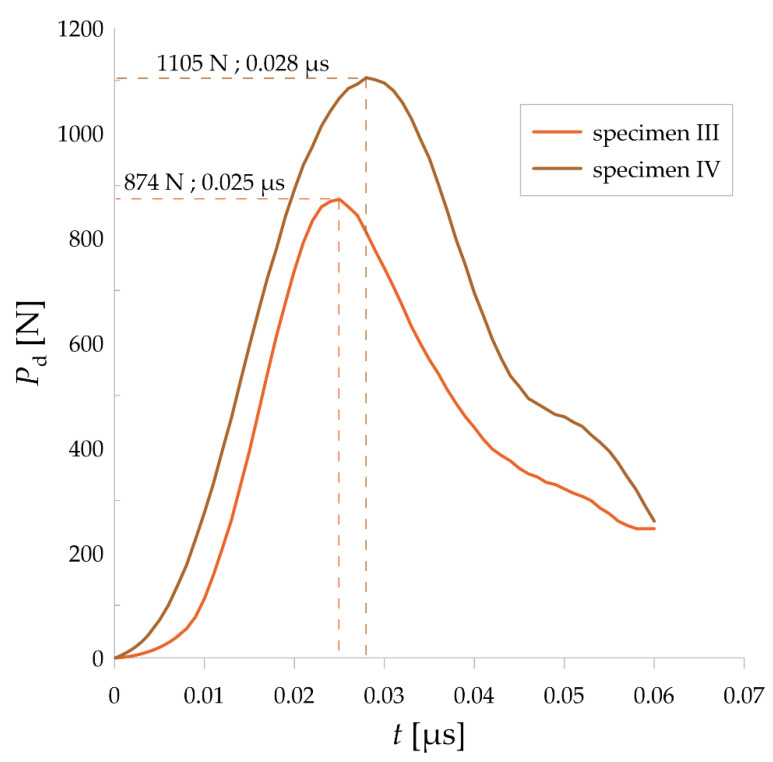
Time-courses of dynamic forces for the two tested SM25T specimens.

**Figure 5:**
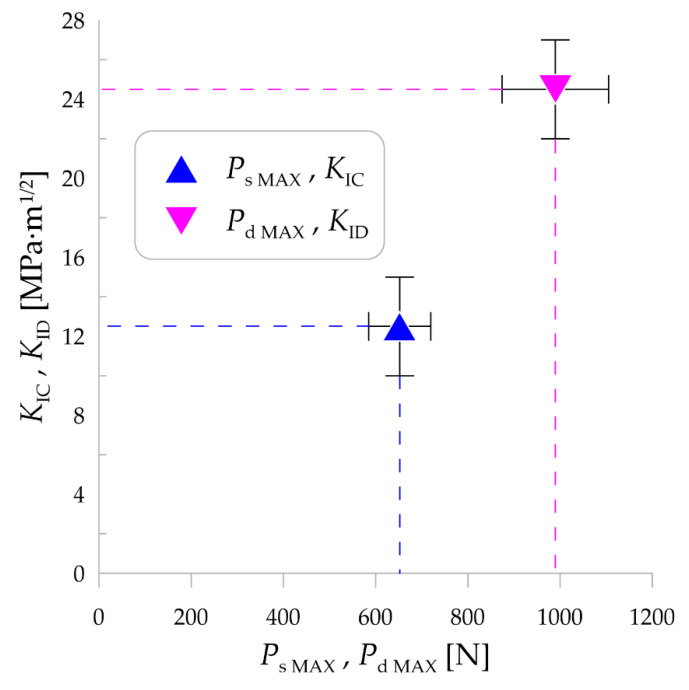
Mean values of static and dynamic fracture toughness (*K*_IC_ and *K*_ID_, respectively) versus static and dynamic force (*P*_s_ and *P*_d_, respectively).

Values of static force versus displacement were plotted in Figure 3 for the two selected samples. It is clearly visible that the maximum forces were at comparable level in both cases, as well as that the curves go in the similar way either. Moreover, the displacement was equal approximately 0.18 mm. Further, time-courses of dynamic forces for these specimens ran similarly, as shown in Figure 4. The mean values of maximum static and maximum dynamic force for the set of SM25T specimens were equal to 652 N and 989.5 N, respectively. These values were set together with the mean values of static fracture toughness and dynamic fracture toughness as indicated in Figure 5. Mean values of *K*_IC_ and *K*_ID_ were equal to 12.5 MPa·m^1/2^ and 24.5 MPa·m^1/2^, respectively. It is noticeable that *K*_ID_ was approximately two times higher, which is correlated with the differences in maximum values of static and dynamic force. As expected, it is clearly visible that fracture toughness values for examined SM25T specimens were much higher than in case of epoxy specimens, which were the point of Authors’ previously taken study [28]. Based on that, the highest values of static and dynamic fracture toughness for cured epoxy resin was equal to 2.42 MPa·m^1/2^ and 3.09 MPa·m^1/2^, respectively. The above described comparison was made to justify the reliability of examination method and to emphasize the scale of difference for brittle materials (epoxy resins) in comparison to WC.

It can be noticed that the value of static fracture toughness in the case of SM25T specimens was at a comparable level as the highest values of *K*_IC_ for cemented WC described in [11,12,13,14]. It can be concluded that in the case of WC-based materials methods of their preparation plays a significant role in changing of mechanical properties. Specimen preparation technique that was applied (and described in Section 2) led to obtain the material with relatively high *K*_IC_ in comparison with cemented carbides. However, different examination procedures were taken to obtain *K*_IC_ values in this study than in [11,12,13,14], where Vickers indenter was used. Thus, it would be interesting to test cemented carbides in the same way which could cause better comparison of the results. Furthermore, the values of dynamic fracture toughness in the case of SM25T specimens are higher than static and seem to be in a good agreement with [15,16].

**Figure 6:**
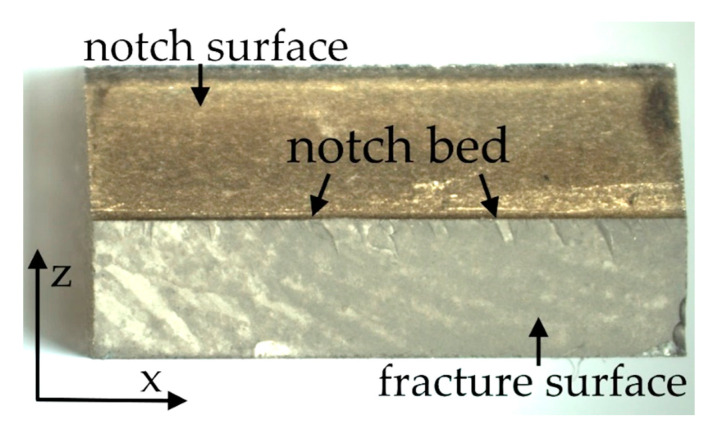
A real photo taken after the break of SM25T specimen.

Moreover, fracture surface analysis was conducted in addition to fracture toughness calculations. The overall view of a fracture surface is presented in Figure 6.

**Figure 7:**
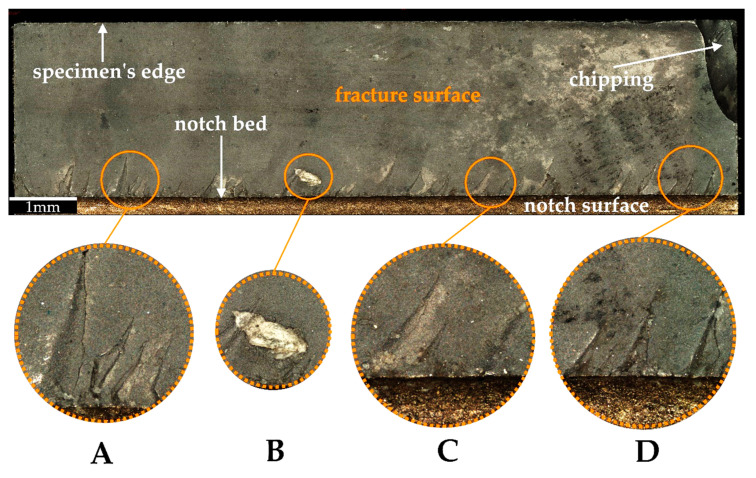
Microscopic view of the fracture surface after impact test with (**A**–**D**) sections indicated.

**Figure 8:**
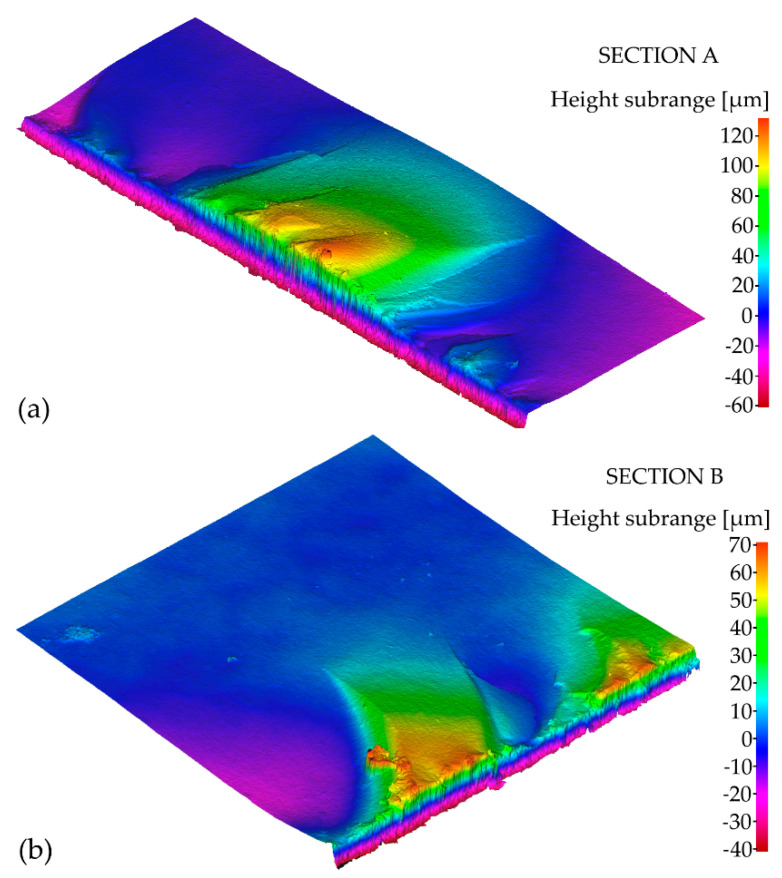
3D contour diagrams for: (**a**) section A; (**b**) section B of the fracture surface after impact test.

**Figure 9:**
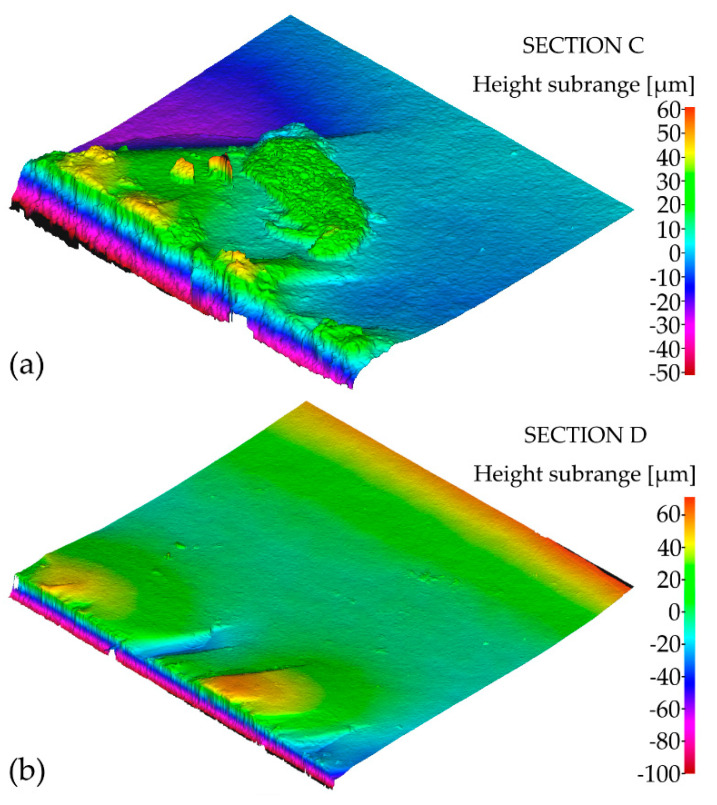
3D contour diagrams for: (**a**) section C; (**b**) section D of the fracture surface after impact test.

Based on the microscopic analysis of the fracture surface after impact test, irregular fracture edge in the region of notch bottom can be observed. Four different sections on the fracture surface were selected and designated as A, B, C, D. Their magnified views are shown in Figure 7. Some specific irregularities, described as “river patterns” are noticeable in sections A, C, and D. They are located at an angle of approx. 7°–9° to the notch bed. The irregularity presented in section B appeared probably due to manufacturing error. In addition, some pores (empty spaces) were found on the fracture surface. Moreover, in Figure 8 and Figure 9 3D contour diagrams are presented which shows surface topography for the four sections with irregularity highness indicated (range of 0–20 µm). Conducted analysis proved that fracture was the brittle one which is confirmed by the presence of “river patterns” as well as the graph presented in Figure 4 where after the dynamic force reaches its maximum value, rapid drop of load appears.

**Figure 10:**
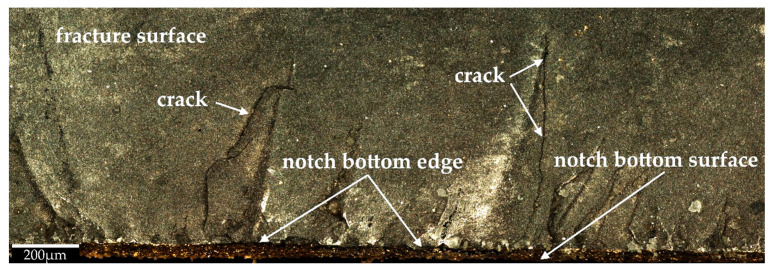
Microscopic view of a fracture surface after impact test with microcracks indicated.

Furthermore, in the middle of fracture surface, short and long microcracks are clearly visible and presented in Figure 10. These cracks have a length up to 70% of *W-a* and they propagate towards z direction (see Figure 2).

**Figure 11:**
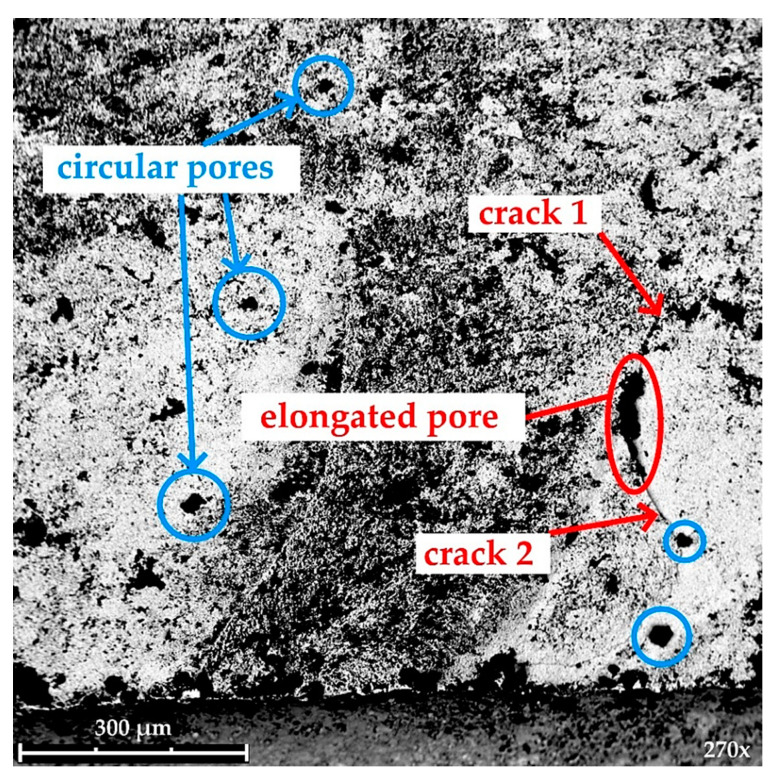
SEM microphotograph of the fracture surface.

As is visible in Figure 11, there is a significant amount of voids (pores) with a wide variety of sizes and shapes. Many of the pores are nearly circular in shape (marked in blue), but many others are elongated in shape—most likely being conglomerates of several voids. This means that the material under research must be considered as porous solid, which is typical for materials produced with the technique of powder sintering. Figure 11 shows something else—the degree of porosity varies in the volume of the material. Namely, the upper and the middle part of the microphotograph is clearly more porous than the bright parts on the left and on the right. As known from the literature [35,36,37], pores are stress concentrators and as such can be crack initiators—see the elongated pore in Figure 11. Moreover, there is a phenomenon of pore coalescence at grain boundaries of polycrystals [38,39], so that cracks are formed through sequential cracking of the thin necks between the adjacent pores—cf. crack 1 in Figure 11. However, this is not the only way of cracks’ formation (ex. crack 2), as in sintered brittle materials shrinkage of the grains in cooling phase of fabrication is different in crystallographic directions. The above short characterization of the fractured WC specimens gives several indications for the forthcoming—more detailed fractographic research, with exploitation of advanced image analysis software.

## 4. Conclusions

The study presented in the current article is an attempt to exploit typical fracture mechanics tests, such as three-point bending performed both in static and dynamic regime for the purpose of quick quality assessment of cutting inserts made of strong but brittle materials such as the tungsten carbide. This way skills scientific of some of the research team members meets the practical needs of the others, working in the field of technology. The above material characterization allows the industrial engineers better understand the reasons for premature wear of the cutting inserts and to program the CNC machines with respect to the maintenance effectiveness.

Based on the conducted study, it can be concluded as follows:

(1) Mean static and dynamic fracture toughness of SM25T specimens is equal to 12.5 MPa·m^1/2^ and 24.5 MPa·m^1/2^, respectively.
(2) The method used to determine fracture toughness of WC-based materials seems to be reliable because the obtained results are within the range of 7-25 MPa·m^1/2^ given in [16] as expected for hard metals.
(3) Microscopic analysis of the fracture surface after impact test shows that during material failure some cracks propagate inside material. In addition, cleavage steps appear which are described as “river patterns” and characteristic for brittle fracture.
(4) Topography analysis of the four sections selected on the fracture surface shows irregularity highness of 0–20 µm.
(5) SEM analysis of the fracture surface shows material porosity which is caused probably by manufacturing technique (e.g., while sintering). Moreover, material porosity is not homogenous on the entire fracture surface.

The tested material itself—WC—turned out to be linear elastic and strong but brittle. Its structure was not free of flaws what is a derivative of the fabrication technology—powder sintering. Even though producers of the WC inserts strive to gain high quality of their products they are unable to evade a certain level of spread of the material’s strength, as this is a permanent disadvantage of brittle materials, having relatively low value of the Weibull modulus. This justifies the “technological” attempts to check at least excerpts of the cutting inserts deliveries regularly. The current study aims at developing an industrial cutting insert quality reporting procedures based on determination of their limit strength in static and dynamic regime. Other specialized test configurations, different from the three-point bending setup, are now considered. The quality assessment procedures will be enriched with a detailed fractographic analyses with a specialized software.

## Data Availability

The data underlying this article will be shared on reasonable request from the corresponding author.
